# Have wind turbines in Germany generated electricity as would be expected from the prevailing wind conditions in 2000-2014?

**DOI:** 10.1371/journal.pone.0211028

**Published:** 2019-02-06

**Authors:** Sonja Germer, Axel Kleidon

**Affiliations:** Biospheric Theory and Modelling Group, Max Planck Institute for Biogeochemistry, Jena, Germany; University College Cork National University of Ireland, IRELAND

## Abstract

The planning of the energy transition from fossil fuels to renewables requires estimates for how much electricity wind turbines can generate from the prevailing atmospheric conditions. Here, we estimate monthly ideal wind energy generation from datasets of wind speeds, air density and installed wind turbines in Germany and compare these to reported actual yields. Both yields were used in a statistical model to identify and quantify factors that reduced actual compared to ideal yields. The installed capacity within the region had no significant influence. Turbine age and park size resulted in significant yield reductions. Predicted yields increased from 9.1 TWh/a in 2000 to 58.9 TWh/a in 2014 resulting from an increase in installed capacity from 5.7 GW to 37.6 GW, which agrees very well with reported estimates for Germany. The age effect, which includes turbine aging and possibly other external effects, lowered yields from 3.6 to 6.7% from 2000 to 2014. The effect of park size decreased annual yields by 1.9% throughout this period. However, actual monthly yields represent on average only 73.7% of the ideal yields, with unknown causes. We conclude that the combination of ideal yields predicted from wind conditions with observed yields is suitable to derive realistic estimates of wind energy generation as well as realistic resource potentials.

## Introduction

With the *Energiewende* or energy transition from fossil fuels to renewables, wind energy became a mainstream energy source [1]. It was the second largest renewable energy source after hydropower in 2015 with a total installed capacity of 433 GW globally [2]. According to the EU Energy Roadmap 2050, apart from energy conservation the switch to renewable energy sources is the second major prerequisite for a more sustainable energy system [3]. In 2016, renewable energy sources had a share of 32% of total electrical energy production in Germany with wind energy contributing about a third to this share [4]. The German legislation plans to further extend renewable energy and sets the target to generate 80% of electrical energy from renewables by 2050 [5]. This extension includes additional installation of 2.9 GW capacity per year for onshore wind energy and an increase of installed capacity in offshore areas to 15 GW by the year 2030.

In order to understand if the target of renewable energy generation can be met with such an increase in installed capacity requires estimates for the performance of wind turbines, knowledge about the factors influencing wind energy generation over time, and to the extent to which wind energy generation can be estimated from large-scale meteorological datasets of wind fields. Turbine performance depends on wind speed distribution and direction, which can vary strongly from day to day due to changes in synoptic activity in the atmosphere, and on air density, which has a comparably much weaker variation. In the long-term, factors which potentially decrease wind speed can have a negative effect on wind energy production [6], such as climate change and changes in surface roughness due to land-use change [7] or the atmospheric effects of large-scale wind energy use [8–11]. It is well known that a concentrated arrangement of wind turbines in wind parks leads to wake effects, reducing energy yield for those turbines standing in the wake of others [12–14]. An increase of average wind park size with time in a region can thus decrease the average performance of its wind turbines as well. In addition, the energy output of wind turbines can be affected by feed-in management that reduces or stops energy feed-in due to insufficient grid capacity, e.g. during periods of high wind speeds. Wind park performance can also decrease due to ageing effects of turbines and increased downtimes toward the end of their lifetime [15]. Such impacts on wind energy yields have received little attention in the past. The age effect has been quantified for selected wind turbines in Sweden [16] and for wind parks in the UK, but not for single wind turbines [15]. Such estimates for wind parks include effects of early turbine death, increasing artificially the average effect of ageing itself. Furthermore there are no country-wide estimates of the size of wind energy losses due to ageing or due to changing wind park sizes with time. Usually, the capacity factor of wind turbines, i.e. the ratio of actual energy generation to the capacity of the turbine, is determined to obtain such estimates and to compare the performance of different wind turbines or track their performance over time. However, the capacity factor also depends on wind availability, which can change from year to year, as well as the specific power of turbines, that is, the ratio of turbine capacity to rotor swept area. Hence, the capacity factor is only one aspect that characterizes country-wide performance of wind turbines, and only if the mean specific power of all wind turbines does not change over time.

Our aim in this paper is to evaluate the role of these mechanisms that lower turbine yields in observed wind energy generation in Germany for the years 2000 to 2014. To do so, we use a dataset of wind conditions in combination with turbine characteristics to estimate the yield that can be expected for the turbines in the ideal case of no such negative effects. We then use this estimate in combination with a dataset of reported yields of a subset of wind turbines in Germany to attribute deviations from this ideal case to the influence of turbine age, park size and regional installed capacity. We then apply a statistical model that includes these influences to predict turbine efficiencies and wind energy generation for all wind turbines in Germany. We discuss the outcome of this statistical analysis in terms of the different factors that reduce turbine yields. We close with a brief summary and conclusions.

## Methods

### Data sources and preparation

We used German monthly energy yield data on a turbine basis from the 'operator database' (http://www.btrdb.de/, [17]) and related them to energy yield predicted on the basis of wind speed and air density calculated from the reanalysis dataset COSMO-REA6 provided by Germany's National Meteorological Service (DWD, Hans Ertel Centre for Weather Research, https://www.herz-tb4.uni-bonn.de, [18]).

### Operator database

The operator database includes the location of German onshore wind turbines since 1988 and for a subset of turbines the monthly energy yields. This is the only publicly available database of wind energy yields per turbine in Germany. The database consists of a site and a yield table. The site table (here referred to as “BDB_sites_”) consists of information provided by manufacturers and operators. It includes the location of the turbines in terms of the postal code of the area as well as the manufacturer, capacity, hub height, rotor diameter, and the month of start and end of operation. The BDB_sites_ database does not report exact positions of wind turbines and therefore the relative position of wind turbines in wind parks to the predominant wind direction is unknown. At the end of December 2014, the total installed capacity of 25296 turbines registered in the database was 37.6 GW, which is within the range of reported values of 36.6 GW [19] and 40.5 GW [20].

A selected group of wind turbine operators voluntarily report monthly wind energy yields for about 25% of all wind turbines in Germany, which is continuously added to the yield table of the operator database. The yield dataset used in this study only included time series of monthly energy yields of at least five years' length. To identify the effect of wind farm size, we considered yields only for those months in which all turbines in the wind park were in operation. In addition, yields of months including shut down periods of turbines due to maintenance or other reasons as well as yields after wind park extensions were excluded. This procedure excluded reported output after re-powering. The final yield dataset (here referred to as “BDB_yield_”) included 5498 turbines with a total of 261012 monthly energy yield data entries reported from January 2000 to December 2014. While 531 turbines were single turbines, the rest was grouped in 921 wind parks. We will refer to the reported monthly wind energy yield as "actual turbine yields".

### Climate database

To estimate monthly energy yields from wind and turbine characteristics for all turbines in Germany listed in the BDB_sites_ dataset, we used the regional reanalysis COSMO-REA6 dataset provided by the DWD’s Hans-Ertel-Centre for Weather Research [18]. This reanalysis included the assimilation of observations from weather stations, including 10 m wind speeds, so that trends in wind speeds [7] should be accounted for. The spatial resolution of COSMO-REA6 is about 6.8 km and hourly values of wind fields are available. The study period covered years with contrasting wind conditions. While the frequency of high wind speeds at 100 m height above the ground was comparatively high in the years 2007 and 2008, it was low in the years 2004 and 2014 (S1 Fig). On average over all of Germany, the wind speeds at 100 m height decreased by -0.017 m/s per year during our study period (S2 Fig), which is close to the mean trend of reported values reviewed by McVicar et al. [21].

The climate data analysis was performed with the Climate Data Operators (CDO) software of the German Climate Computing Center (DKRZ, https://code.mpimet.mpg.de/projects/cdo/). The data was transformed to a regular grid in order to combine it with the turbine data available on a basis of postal codes. The wind speed data was taken from the three lowest model layers and interpolated to the hub heights of turbines and then the mean wind speed *v* of the postal code area was calculated in which the turbine is situated in. Equivalently we calculated the air density *ρ* from temperature fields and surface pressure.

To calculate the expected yield from these meteorological conditions, we used an idealized power curve and combined it with the attributes of the wind turbines in terms of hub height, rotor diameter *d*, and turbine capacity *P*_*max*_. We assumed no generation for wind speeds less than *v* = 3.5 m/s. For greater wind speeds, we calculated the electricity generation rate, *P*_*e*_ (in W) through the rotor-swept area *A* (= π (*d*/2)^2^) of the turbine by:

$$P_{e}=\eta \frac{\rho }{2}v^{3}\;A$$ (1)

We assumed a power coefficient of *η* = 44%, a typical value for a wide range of turbines (The collection of turbine data shown in Carrillo et al. [22] give a distribution of the power coefficient with an interquartile range of 43–46%, see S3 Fig). We used a generic value because for a number of turbines, no specific information on the power coefficient could be obtained, either because the manufacturer does not provide this information, or because the manufacturer no longer exists or was bought up by another manufacturer. Variations in the power coefficient affect the estimate in an approximately proportional way, so that a power coefficient of 40% yields about 10% less generation (S4 Fig).

We further limited electricity generation to the capacity of the turbine at high wind speeds (i.e., *P*_*e*_ ≤ *P*_*max*_), and used a cut-out wind speed of *v* = 25 m/s, assuming that wind turbines are switched off at such wind speeds and above (note, however, that such wind speeds are practically absent in Germany, see S1 Fig).

The turbine characteristics of the BDB_sites_ database enter this estimate through the hub height, which was used to interpolate wind speeds from the COSMO-REA6 dataset, rotor diameter was used to determine the rotor-swept area *A*, and the turbine capacity was used to limit electricity generation at high wind speeds.

Electricity generation was calculated for each wind turbine for each of the hourly wind speed data and this estimate was aggregated to the monthly time scale. We refer to this estimate as the “ideal turbine yield” as it sets a reference without yield decreasing effects.

### Data preparation

As a measure of turbine performance, monthly capacity factors were calculated by dividing actual energy yield per month by maximum possible energy yield per month (the installed capacity times hours per month). Capacity factors were also calculated from the estimated ideal turbine yield derived from climate reanalysis data (CF_ideal_) and from actual turbine yield in the BDB_yield_ table (CF_actual_). To assess the regional effect of installed capacity on energy yield per turbine, the total installed capacity per postal code area and month was calculated using the BDB_sites_ table. In 2014, wind turbines operated in 2328 out of 8199 postal code areas in Germany.

**Fig 1:**
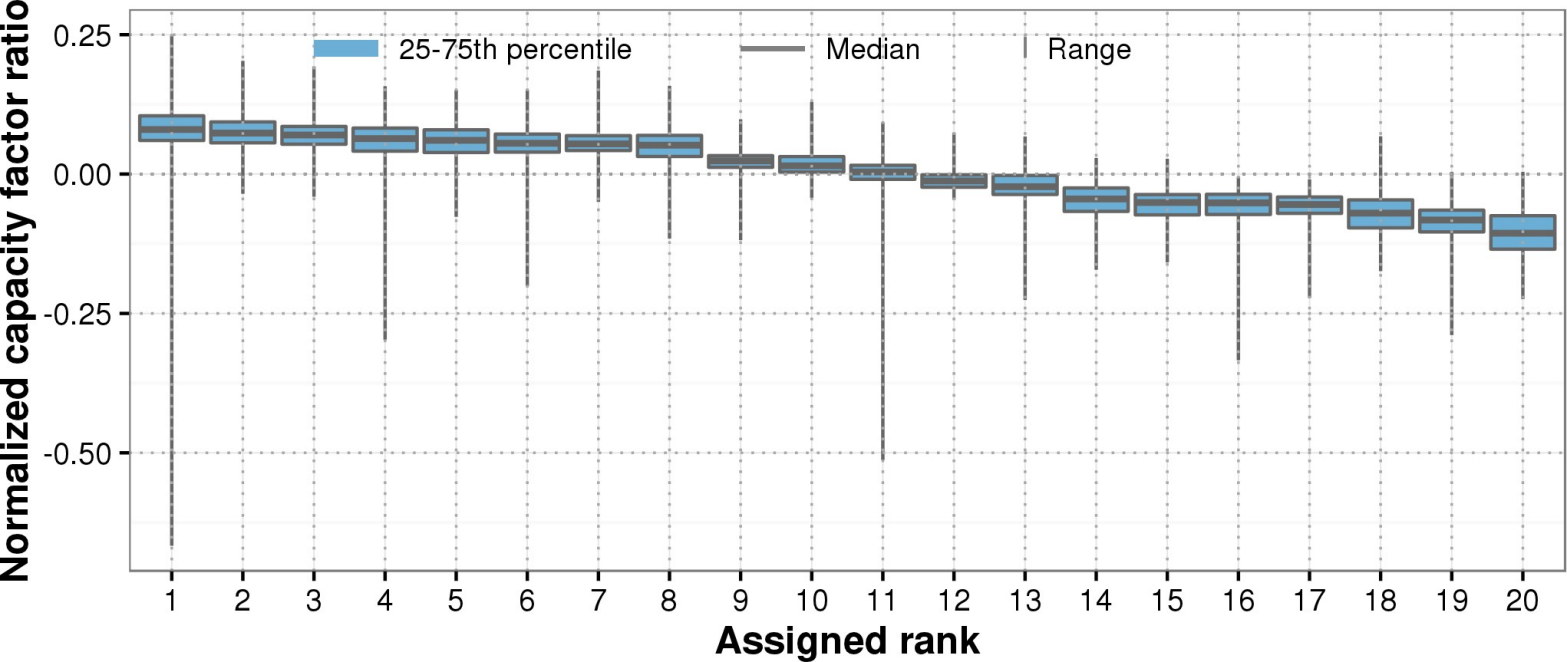
Example of assigned ranks for one wind park of 20 turbines with energy yield data covering 10 years. The ranks were assigned according to the median ratio of ideal and real capacity factors normalized by their median per month and park.

The age was calculated for each month and turbine in BDB_sites_ in decimal years. To estimate the wake effect in wind parks, we assigned a rank to each turbine in a park that we identified through the ID fields in the dataset. For each turbine in the park, we calculated a ratio of capacity factors each month, CF_actual_ divided by CF_ideal_, and normalized them by their median per month and park. Then, for each park, turbines were ranked by their median normalized capacity factor ratio. An example for a single wind park is shown in Fig 1. The normalization was performed in order to eliminate seasonal variability. As a result, the interquartile ranges of monthly normalized capacity factor ratios per turbine are very narrow (see e.g. Fig 1), suggesting that significant differences in yield associated with the turbines in the park and that these differences did not change substantially over time.

### Data analysis

All statistical modelling and prediction as well as graphics were done using the program R, version 3.2.2 [23], incorporated in RStudio, version 1.0.153 [24].

**Fig 2:**
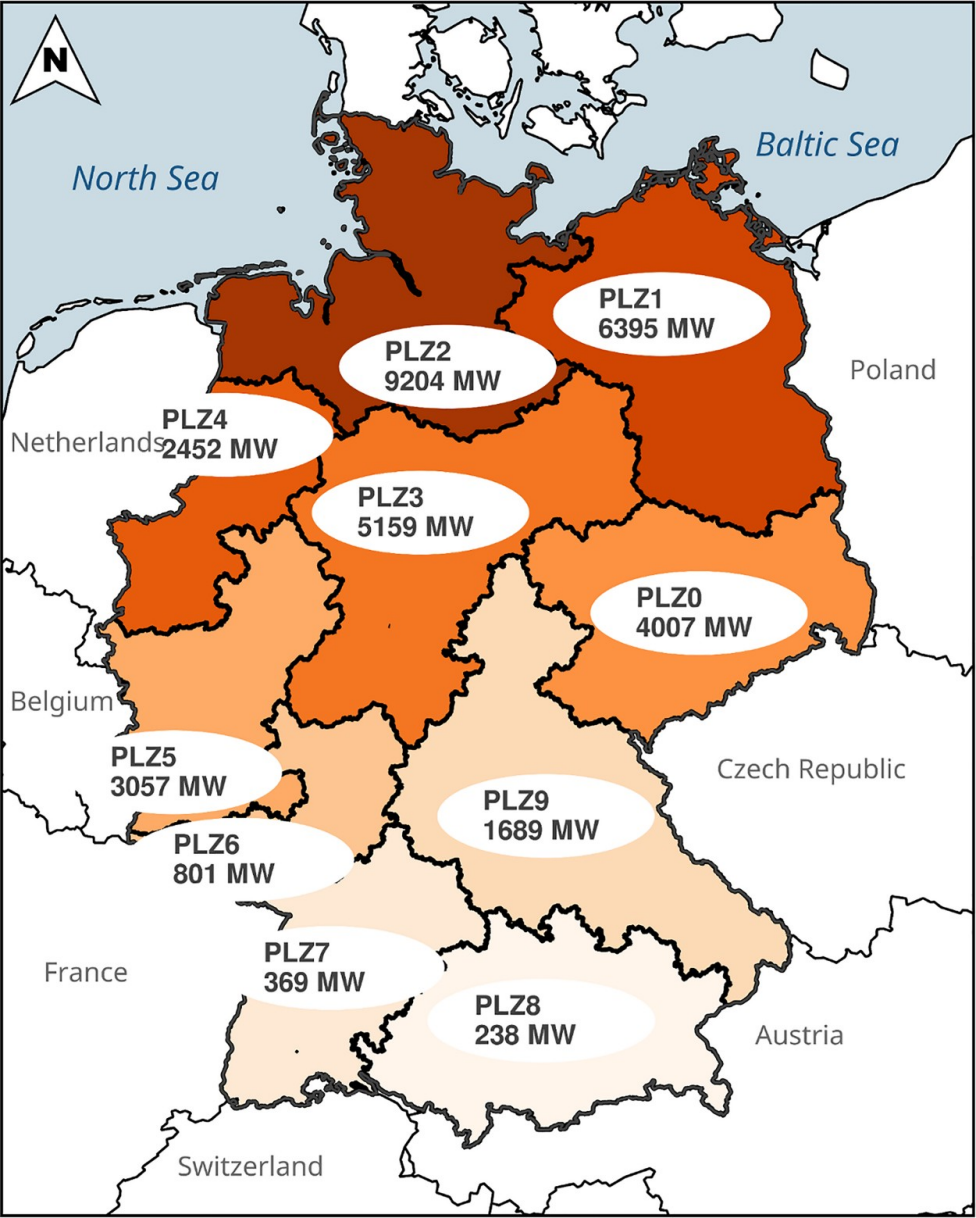
Map of Germany's 10 main postal code zones (PLZ) and their total installed capacity of wind turbines in December 2014. Main postal code zones are divided in up to a maximum of 1000 postal code areas. Seven zones were included as fixed effect in the model: PLZ0 to PLZ5 and PLZ6+. The latter includes PLZ6 to PLZ9 as in the south of Germany the installed capacity is low.

The ultimate aim of the data analysis is to estimate energy yields. In order to avoid the influence of seasonality we opted to estimate first the capacity factors and use them afterwards to calculate energy yields. A linear mixed-effects model was set up to assess the effects on capacity factors from different independent variables simultaneously. This approach is based on capacity factors calculated from actual turbine yields (*CF*_*actual*_) and from ideal turbine yields (*CF*_*ideal*_). As yields were reported each month per turbine, their observations and residuals are not independent. Each turbine in the dataset had a unique identification number (*ID*). To control for non-independence of residuals, the *ID* of each turbine was treated as random effect in the model. As fixed effects, turbine age (*AGE*) and turbine rank (*RANK*) were included. In addition, main postal code zones (*PLZ*, Fig 2) were included as fixed effects, as visual data analysis suggested the existence of regional differences. Data visualization also indicated that the age, rank and postal code zone effects depended on the average capacity factors of turbines. The general model formulation was:

$$\begin{array}{l} CF_{actual,i,m}∼\beta_{0}+\beta_{1}CF_{ideal,i}+\beta_{2}\left(CF_{ideal,i}\times AGE_{i}\right)+\beta_{3}\left(CF_{ideal,i}\times RANK_{i}\right)\\ \;+\beta_{4}\left(CF_{ideal,i}\times I{\left[PLZ0\right]}_{i}\right)+\beta_{5}\left(CF_{ideal,i}\times I{\left[PLZ1\right]}_{i}\right)\\ \;+\beta_{6}\left(CF_{ideal,i}\times I{\left[PLZ2\right]}_{i}\right)+\beta_{7}\left(CF_{ideal,i}\times I{\left[PLZ3\right]}_{i}\right)\\ \;+\beta_{8}\left(CF_{ideal,i}\times I{\left[PLZ4\right]}_{i}\right)+\beta_{9}\left(CF_{ideal,i}\times I{\left[PLZ5\right]}_{i}\right)+b_{0,m}\\ \;+b_{1,m}CF_{ideal,i,m}+\varepsilon_{i,m} \end{array}$$ (2)

where *β*_0_ is the common intercept, *β*_*1*_ is the slope of *CF*_*actual*_ and *CF*_*ideal*_, *β*_*2*_ to *β*_*9*_ are changes of *β*_*1*_ induced by the single fixed effects. The index *i* denotes the *i*th observation and index *m* the *m*th subject (i.e., turbine *ID*). The parameters *b*_0_ and *b*_1_ are the random intercept and slope, respectively, which vary with turbine *ID*, while *ε* is the error term. The variables *AGE* and *RANK* were centered around their mean. The variable *I[*.*]* is a dummy variable representing the level of the factor postal code zones (*PLZ*). For instance, *I[PLZ2]* is a dummy for postal code zone 2. The model uses the mean of all postal code zones as a reference. As a slope is estimated for the reference model, dummy variables are needed only for 6 out of 7 postal code zones. The mixed-effects model was fitted using the “lmer” function from the “lme4” package [25] with maximum likelihood parameter estimation (lme4 notation: *lmer(CF*_*actual*_ *~ CF*_*ideal*_ *+ CF*_*ideal*_:*AGE + CF*_*ideal*_:*RANK+ CF*_*ideal*_:*PLZ + (CF*_*ideal*_ *|ID)*, *data = dataset_name*, *contrasts = list(PLZ = contr*.*sum)*, *na*.*action = na*.*exclude*, *REML = F))*. Normality and homogeneity of variance were tested by examining the normal qq-plots and the residuals versus fitted-values plots, respectively. Regional installed capacity was not included as fixed effect in the model as it showed to be a poor predictor when analysing data subsets by the fixed-effects model approach and because of high collinearity of it with the *AGE* predictor as an increase of installed capacity per postal code area involves an increase of age of existing turbines.

The prediction of capacity factors for all turbines not included in the BDB_yield_ dataset and for months in which yield data is not available was done by using the parameters estimated by Eq 2, which were used in the R function “predict.merMod”. This function uses the fitted mixed-effects model for the prediction of new values. For the mixed-effects model the random effects per turbine could only be estimated for the turbines in the BDB_yield_ data, but not for those only present in the BDB_sites_ data. Therefore, all predictions were performed without including the random effect. Annual yields were calculated from predicted capacity factors and compared to reported electricity generation by wind energy in Germany [26].

## Results

### Wind turbine characteristics in Germany from 2000 to 2014

**Fig 3:**
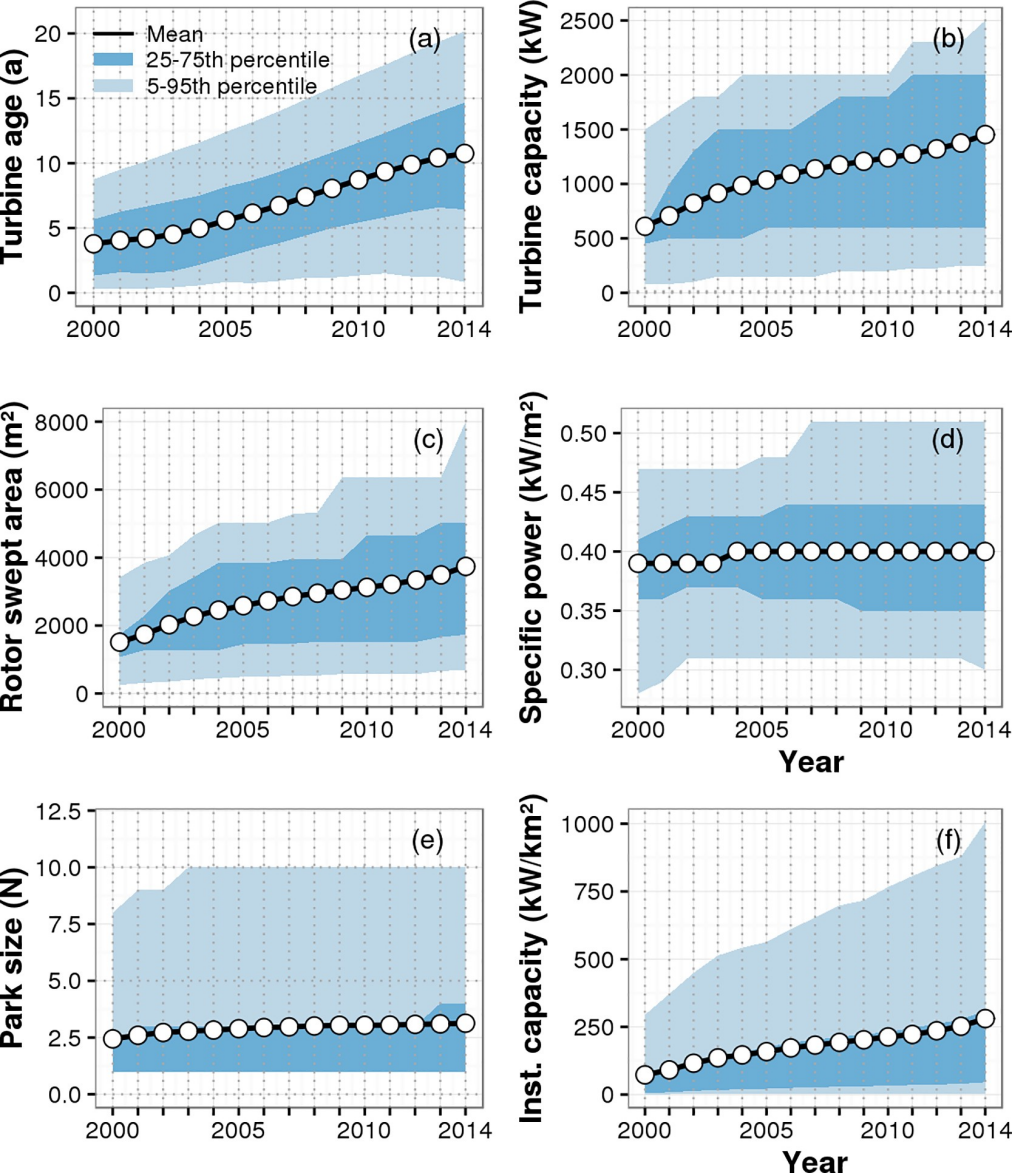
Changes in onshore wind turbine characteristics in Germany from the year 2000 to 2014. The panels show (a) the age of wind turbines, (b) turbine capacity, (c) rotor swept area, (d) specific power and (e) park size (in terms of N, the number of turbines per park) and (f) the density of installed capacity per postal code area. The black lines refer to the mean of the distribution of values, while the shaded areas indicate the range of values in terms of the 25%-75% percentile (dark blue) and the 5%-95% percentile (light blue).

The overall trends in wind turbine characteristics in Germany for the years 2000 to 2014 that are directly calculated from the BDB_sites_ database are shown in Fig 3 (see S1 and S6 Tables for percentile values shown in Fig 3). While some characteristics of wind turbines or parks changed, other remained rather constant over time. The mean turbine age in the year 2000 was only 3.8±2.7 (± SD) years and it increased to 10.8±5.8 years in 2014 (Fig 3A). While in the year 2000, the mean turbine capacity was 611±401 kW, it increased to 1453±808 kW in the year 2015 (Fig 3B). The mean rotor swept area also increased from 1513±899 m^2^ to 3742±2237 m^2^ within this time period (Fig 3C). Mean turbine capacity and mean rotor swept area increased almost at the same rate of 2.4 and 2.5 from 2000 to 2014. As a result, the mean specific power, which is the ratio of turbine power to rotor swept area, increased only from 0.39±0.05 kW m^-2^_r_ in 2000 to 0.40±0.06 kW m^-2^_r_ in 2014 (Fig 3D). During the same time period the mean size of individual wind parks increased only slightly from 2.4±6.2 to 3.1±5.4 turbines per park (this includes single turbines with a park size of 1 turbine, Fig 3E). Mean installed capacity per postal code area, however, increased by a factor of 2.6 from 216±342 to 564±656 kW/km^2^ (Fig 3F).

**Fig 4:**
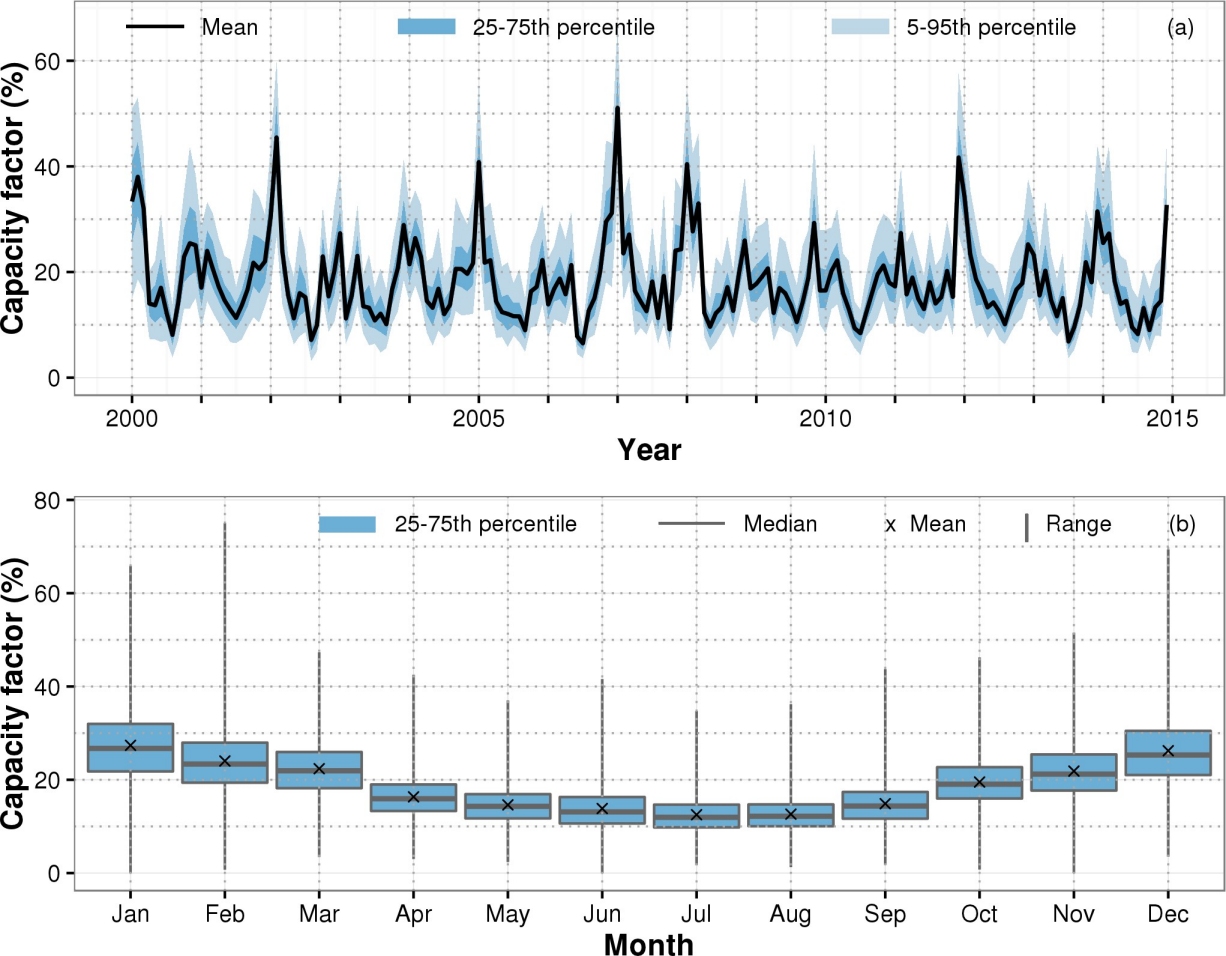
**Temporal variability of capacity factors illustrated as (a) time series of monthly capacity factors of all turbines in the BDB_yield_ database and as (b) seasonal variability of monthly mean capacity factors per turbine**.

The mean monthly capacity factor calculated from the BDB_yield_ database fluctuates seasonally between 10 and 30% reaching values above 40% only at a few times in winter (Fig 4). The long-term mean capacity factor is 18.3±7.5%.

### Turbine performance by climate driven estimates

The mixed-effects model approach assessed effects of age and rank in wind parks simultaneously. The model regresses capacity factors derived from reported monthly yields (CF_actual_) over those calculated from climate based estimates (CF_ideal_). The estimate for *β*_*1*_ CF_ideal_ of 0.7372±0.0020 (Table 1) represents the slope of CF_actual_ over CF_ideal_ at age zero and rank one and it is the mean of all postal code zones. Thus, all turbines with reported actual monthly yields on average generate only 73.7±0.2% of what has been estimated in the ideal case from wind conditions and turbine characteristics. Note that the given uncertainty only includes the uncertainty of the slope estimation by the statistical model and does not include possible uncertainties of the wind fields, the reported yield and the idealized power curve. Turbine age and rank in the wind parks as well as the postal code zones had a significant influence on the monthly capacity factors per turbine (Table 1). The slope of CF_actual_ over CF_ideal_ and, hence, the deviation from the ideal case decreases by 0.63±0.01% per year of turbine age and by 0.49±0.02% per turbine rank.

**Table 1:** Estimated fixed effects, their standard errors (SE), and the t-values obtained from the mixed-effects model.

Parameter	Estimates	SE
Intercept (*β*_*0*_*)*	-0.0013**	0.0003
CF_ideal_ (*β*_*1*_*)*	0.7372***	0.0020
CF_ideal_:AGE (*β*_*2*_*)*	-0.0063***	0.0001
CF_ideal_:RANK (*β*_*3*_*)*	-0.0049***	0.0002
CF_ideal_:PLZ0 (*β*_*4*_*)*	0.0751***	0.0046
CF_ideal_:PLZ1 (*β*_*5*_*)*	-0.0413***	0.0034
CF_ideal_:PLZ2 (*β*_*6*_*)*	-0.0727***	0.0028
CF_ideal_:PLZ3 (*β*_*7*_*)*	-0.0224***	0.0037
CF_ideal_:PLZ4 (*β*_*8*_*)*	-0.0588***	0.0048
CF_ideal_:PLZ5 (*β*_*9*_*)*	0.0288***	0.0051

Significance level

**P<0.001

***P<0.0001

degree of freedom = 255330 for all parameters.

The estimates of the postal code zones represent deviations from the mean slope in the respective zone (Table 1). The deviation of slope in region PLZ6+ is 0.0913 as the sum of all deviations from the mean slope needs to equals zero (Table 1). Hence, the difference between actual and ideal turbine yield is greater in Northern Germany (postal code zones 1 to 4) where wind speed and the regional installed capacity are higher than in Southern Germany (zones 0, 5 and 6+, Fig 2, Table 1).

### Energy yield and absolute losses

**Fig 5:**
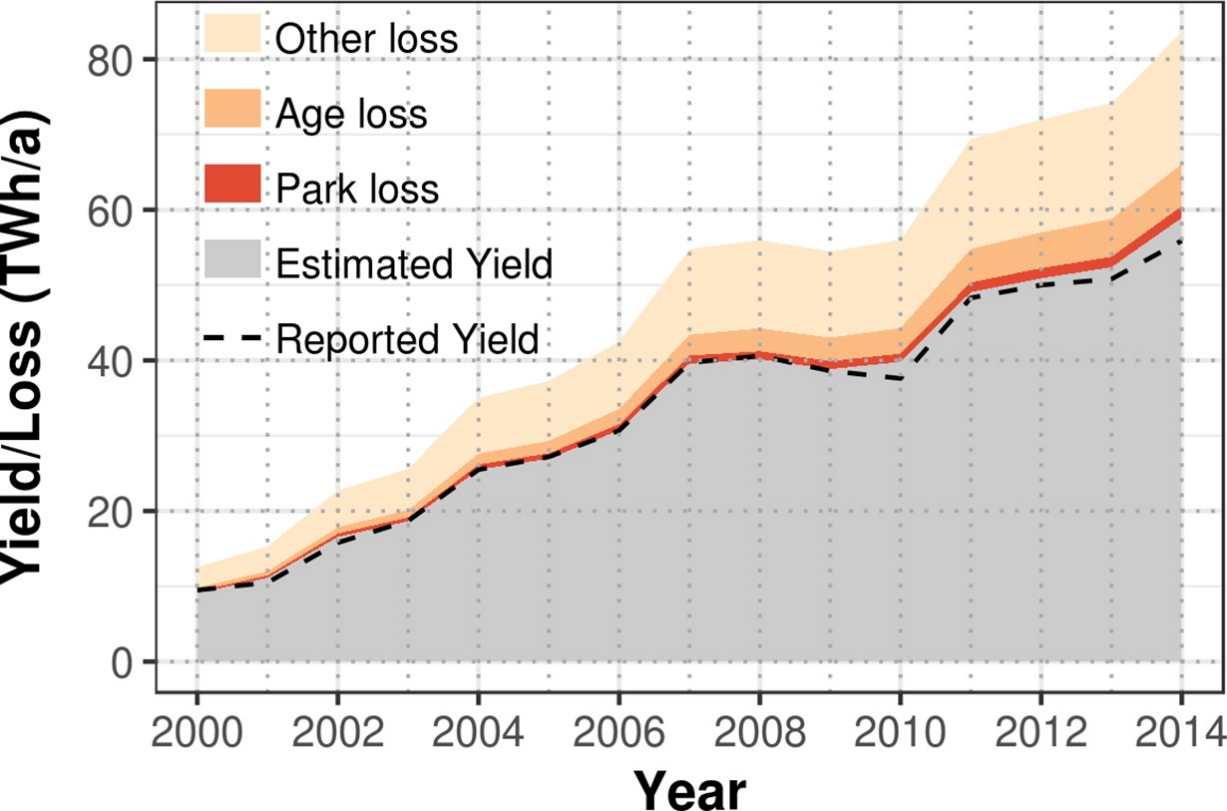
Reported and estimated annual yield for onshore wind energy generation in Germany. The sum of estimated yields and losses due to the age effect, park effect and other effects equals the annual ideal wind energy yield as estimated from wind speed, air density, and turbine characteristics (source of reported yields: [24]).

We next used the estimated parameters of the mixed-effects model and applied it to estimate yields of all wind turbines in Germany to predict the countrywide generation of wind energy. Estimated annual yields increased from 9.1 TWh in the year 2000 to 55.9 TWh in the year 2014 (Table 2, Fig 5). These estimates are very close to the reported values by the German Ministry of Economy and Energy [26]. Using our mixed-effects model, we can quantify two types of losses from ideal to estimated yields. The first type of loss (“other losses” in Fig 5) is related to the slope of CF_ideal_ to CF_actual_. It should be noted that the slope is a result of the statistical model, but we still call it “other loss due to unidentified effects” because the slope itself does not explain the loss, and it could also reflect biases in the wind fields dataset (see discussion below). The second type of loss is related to the age and park effects, which influence the magnitude of the slope. Therefore, “other losses” is the difference between the ideal yield and the estimated yields for the case that all turbines were new and no wake effects between turbines would occur.

**Table 2:** Reported and estimated annual wind energy yields as well as relative losses of energy yield due to effects of age, park size, and other unknown effects. The estimated annual energy yield corresponds to the sum of monthly yields of all turbines in BDB_sites_. Monthly yields of the turbines were derived from capacity factor predictions of the mixed-effects model.

Year	Annual yield (TWh)		Losses (% of ideal yield)			Mean annual capacity factor (%)	Installed capacity in December (GW)
	Reported^1)^	Estimated	By age effect	By park effect	Other effects		
2000	9.5	9.1	3.75	1.99	21.67	0.22	5.7
2001	10.5	11.1	3.79	1.96	21.45	0.19	8.2
2002	15.8	16.6	3.70	1.94	21.37	0.20	11.4
2003	18.7	18.6	3.87	1.91	21.39	0.17	13.9
2004	25.5	25.5	4.08	1.91	21.18	0.19	16.0
2005	27.2	27.0	4.43	1.91	21.32	0.18	17.9
2006	30.7	30.7	4.75	1.91	21.03	0.18	20.1
2007	39.7	39.6	5.06	1.92	20.73	0.21	21.8
2008	40.6	40.2	5.41	1.91	20.93	0.20	23.4
2009	38.6	38.9	5.75	1.91	21.00	0.18	25.2
2010	37.6	39.8	6.09	1.91	20.91	0.17	26.6
2011	48.3	49.1	6.41	1.87	21.00	0.20	28.3
2012	50.0	50.9	6.58	1.86	20.85	0.19	30.5
2013	50.8	52.4	6.77	1.85	20.77	0.18	33.2
2014	55.9	58.9	6.70	1.85	20.92	0.18	37.6

^1)^ [26]

While the loss by the park effect stayed rather constant at 1.9% of total annual energy yield in Germany, the loss by the age effect increased from 3.6% to 6.7% (Table 2, Fig 5). For 2014, the absolute loss for the age and park effect reached 5.6 and 1.6 TWh, respectively. “Other losses” due to unidentified effects led to 71% and 79% of total loss in 2000 and 2014, respectively. Relative to the ideal energy yield, 20% generation losses were due to unidentified effects for all years studied. During the study period the installed capacity in Germany increased by a factor of 6.6 from 5.7 GW in the year 2000 to 37.6 GW in 2014. The annual average turbine performance, however, did not change as indicated by rather constant mean annual capacity factors from 2000 to 2014 (Table 2).

## Discussion

### Influence of turbine age, park size and regional installed capacity on turbine performance

We found that turbine age significantly decreased turbine performance by 0.63±0.01% per year. This effect could, in principle, include other external effects as well, such as increased feed-in management with time or wake effects of newly constructed wind parks upwind from the turbines we considered. As the exact positions of wind turbines were not included in the database, but installed capacity increased continuously from 2000 to 2014 (Fig 3F), yields might have decreased due to the wake effect of newly constructed wind parks. This wake effect would then have been included in the overall aging effect. An effect of the extension of existing wind parks on the turbine performance with time was, however, avoided by excluding yield data after extension took place.

Feed-in management decreased total wind energy yields in Germany by only 2.1% in the year 2014 and below 1% before 2014 (EEG in Zahlen 2015, www.bundesnetzagentur.de). Excluding the year 2014 before fitting the mixed-effects model did not change the significance of the age effect. We therefore assume that feed-in management can be neglected as a relevant factor in our analysis.

The decline of wind turbine performance with age estimated by our mixed-effects model is lower than the 1.6±0.2% per year reported by Staffell and Green [15] for UK wind parks. Staffell and Green [15] estimated the ageing effect relative to average capacity factors per wind park that might have led to an overestimation of the ageing effect due to unknown early turbine death in wind parks. Olauson et al. [16] estimated a performance decline of 0.15 percentage points per year for a dataset of energy yield per turbine from Sweden. For new turbines with a capacity factor of 0.25 this results in a performance decrease of 0.6% per year, which is consistent with our result. Assuming that the mixed-effects model approach represents the average age effect for wind turbines in Germany and a turbine design lifetime of 20 years, the performance of wind turbines averaged over their lifetime would be lower by 20/2 x 0.63% ≈ 6.3% compared to a new turbine only due to the age effect. This age effect can be a crucial factor for wind project planning as the cost of wind energy yield is inversely proportional to the capacity factor [27].

The rank assigned to each turbine representing the wake effect in wind parks let to a significant decreased turbine performance of 0.49±0.02% per turbine rank. A wind park with 6 turbines would have an average yield loss due to the wake effect of 6/2 x 0.49% ≈ 1.5%. This estimate agrees well with published estimates. For instance, Kusiak and Song (Table 4 in [28]) estimated onshore wind energy production with a park layout optimization model. For a wind park size of 6 turbines they found a similar wake loss of 1.6%. For large wind parks, however, wake losses can be much greater. In 2014, 1% of all wind parks in Germany had 22 turbines or more. These 46 wind parks should have had an average yield loss of at least 22/2 x 0.49% ≈ 5.4%, which corresponds to the lower end of estimates for large wind parks with different turbine spacing and site climatology of 5–20% [29,30].

Installed capacity per postal code area had no significant effect on turbine performance, so it would seem that wind turbines in Germany do not generally affect regional wind speeds by much. The installed capacity per postal code area might, however, not be the best proxy for regional installed capacity as the postal code areas differ considerably in size. In addition, areas with high installed capacity next to the coast are expected to be less affected as turbines are aligned in rows along the coast. The question of whether the installed capacity is already high enough in some parts of Germany to detect regional effects on turbine performance might be studied in future, but this would likely require more precise information of turbine positions.

### Ideal yield compared to actual yield

In this study, on average 73.7% of the ideal wind energy yield was converted to electrical energy (actual yield), leading on average to 26.3% of overall losses. This considerable reduction may result from several factors. For instance, the reduction may be due to the generic value of 44% of the power coefficient rather than using realistic power curves for the turbines, or it may result from biases in the wind fields that represent a reanalysis dataset rather than observations. Using a power coefficient of, e.g., 40% could reduce the ideal yield by a factor of about 0.91 (40/44), leading to a reduction of overall losses to 17.3%. However, the reduction of 26.3% is in line with other studies. For instance, Pieralli et al. [31] found that electrical losses amounted to 27% of ideal yield for 19 wind turbines installed in 4 wind parks in Germany. According to their study, most of the losses were attributed to variability in wind direction, while 6% of losses were attributed to turbine errors. For the UK, Staffell and Green [15] found an average difference between ideal and actual yield of 24.5%. Furthermore, an independent comparison of the wind fields of the COSMO-REA6 dataset to wind mast measurements in the range of 10 to 116m showed that the wind fields in the dataset were realistic [32]. Hence, this reduction of actual yields by 26.3% compared to the ideal yields is realistic and consistent to previous studies.

The combination of ideal wind energy yields with the mixed-effects model to estimate actual yields of all turbines in Germany resulted in estimates of total actual annual wind energy generation in Germany that are very close to reported ones (Fig 4, Table 2). An increase of installed capacity in Germany from 5.7 GW in the year 2000 to 37.6 GW in 2014 led to strong yield increases from 9.1 TWh to 55.9 TWh, but the average performance of wind turbines in Germany did not increase as one may expect due to technology improvements. This can be explained by the following considerations. If the turbine capacity is high relative to the rotor swept area, then the turbine's specific power is high, but the capacity factor is low in low wind regions. By decreasing the turbine's capacity, the specific power would be decreased and the capacity factor increased without generating more energy. Therefore, an increase in turbine's specific power can lead to decreases in capacity factors that would appear as a performance decrease. However, the average specific power did not change from 2000 to 2014 (Fig 3D), and hence, the lack of expected performance increases needs to have other reasons. From the year 2000 to 2014, total production losses increased due to an increase of average turbine age with time (Fig 3A). Therefore, expected performance increases due to technology improvements have likely been at least partly mitigated by performance decreases due to ageing of wind turbines in Germany.

## Conclusions

We have estimated ideal wind energy yield for Germany for the years 2000 to 2014 using datasets of wind speed and air density as well as turbine characteristics. We then used the ideal wind energy yield and actual monthly energy yields of a subset of wind turbines to set up a mixed-effects model that we then applied to all wind turbines in Germany to estimate the actual monthly energy yield. Annual sums of actual plus estimated yields of all turbines in Germany were very close to reported ones. On average, however, only 73.7±0.2% of the ideal wind energy yield was converted to electrical energy (actual yield).

For the years 2000 to 2014 the average specific power of all turbines in Germany did not change, so that the capacity factor can be used as a measure of turbine performance. The mixed-effects model indicated that turbine performance was significantly influenced by turbine age and park size. On average, wind parks in Germany lose 6.3% total yield when assuming an average turbine lifetime of 20 years. The ageing effect, as defined here, might, however, include other effects such as changes in maintenance quality, wake effects of newer wind parks, and decreases due to feed-in management or other external factors that decrease wind speed. The park effect decreased total onshore wind energy yield in Germany by 1.9% per year. This share stayed constant over time as did the average park size. Even though the age and park effects led to considerable energy generation losses, the loss due to other unidentified effects led to over 70% of total losses.

The knowledge we gained about the share of ideal and actual energy yield as well as effect of turbine age and park size, should be valuable for the prediction of future energy yields. Assuming that a change in specific power of turbines does not influence the magnitude of the ageing and wake effect, the mixed-effects model might be used together with ideal energy yield estimations to estimate future wind energy yields for different renewable energy scenarios. Specifically, this mixed-effects model would allow to evaluate such scenarios with respect to the increase of installed capacity, the effects of turbine ageing, as well as the effects of park sizes. Such an application of the mixed-effects model to future scenarios would, however, benefit from first assessing its capacity to predict the yield for a set of recently installed and modern turbines with known yields.

## Supporting information

S1 Fig Frequency histograms of hourly wind speeds in Germany for the years 2000 to 2014 (“All”) and for single years within is period extracted from the COSMO-REA6 dataset at 100 m height. The median (solid line), mean (dotted line) and the interquartile range (blue area) for the histogram of the entire period are also shown.
(PDF)

S2 Fig Histogram of the trend in mean wind speeds using hourly wind speeds in Germany for the years 2000 to 2014 extracted from the COSMO-REA6 dataset at 100 m height. The frequency refers to the number of grid cells of the data set showing the trend of a given magnitude. The median (solid line), mean (dotted line) and the interquartile range (blue area) for the histogram of the entire period are also shown. At top, the cumulative distribution function is shown.
(PDF)

S3 Fig Distribution of power coefficients taken from the turbine data provided in the review of Carrillo et al. **(2013).** The median (solid line), mean (dotted line) and the interquartile range (blue area) are also shown.
(PDF)

S4 Fig Sensitivity of estimated monthly yield to the power coefficient for the year 2010. (PDF)

S1 Table Values of age distribution (years) shown in Fig 3A. (PDF)

S2 Table Values of installed capacity (kW) distribution shown in Fig 3B. (PDF)

S3 Table Values of rotor swept area (m2) distribution shown in Fig 3C. (PDF)

S4 Table Values of specific power (kW/m^2^) distribution shown in Fig 3D. (PDF)

S5 Table Values of park size (N) distribution shown in Fig 3E. (PDF)

S6 Table Values of installed capacity (kW/km^2^) distribution shown in Fig 3F. (PDF)
