# Supplementary material for: Have wind turbines in Germany generated electricity as would be expected from the prevailing wind conditions in 2000-2014?
Source: PLoS One. 2019 Feb 6;14(2):e0211028. doi: 10.1371/journal.pone.0211028 (PMC6364903; doi:10.1371/journal.pone.0211028)
Supplement: S1 Fig — The median (solid line), mean (dotted line) and the interquartile range (blue area) for the histogram of the entire period are also shown. (PDF) [file pone.0211028.s001.pdf]

Supplementary Material to:

Have wind turbines in Germany generated electricity as would be expected from the prevailing wind conditions in 2000-2014?

Sonja Germer, Axel Kleidon

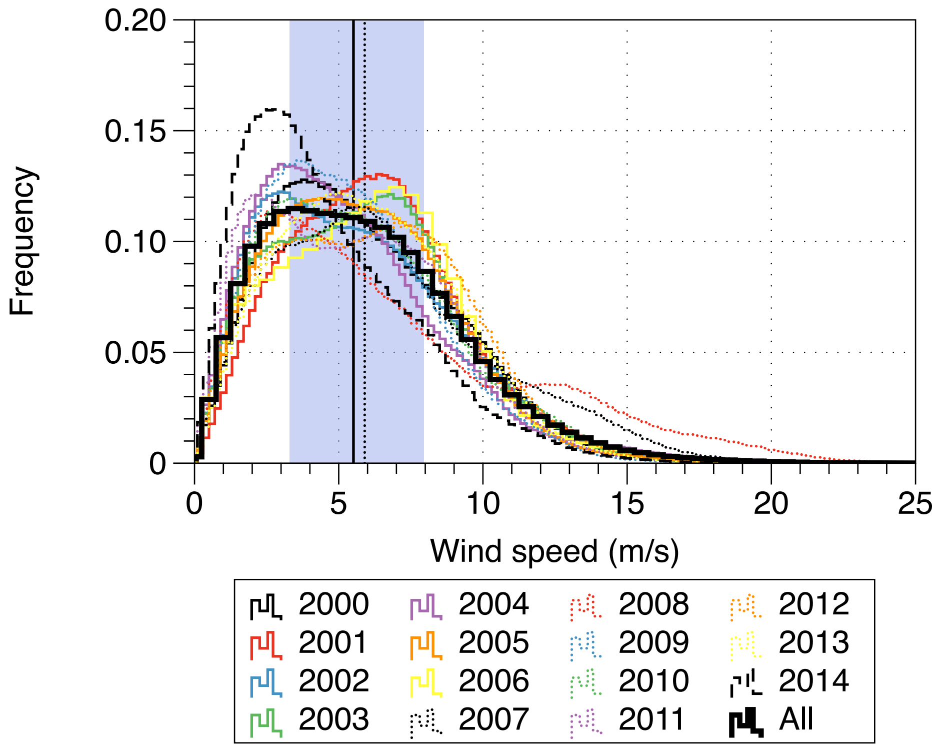

S1 Fig. Frequency histograms of hourly wind speeds in Germany for the years 2000 to 2014 ("All") and for single years within is period extracted from the COSMO-REA6 dataset at 100 m height. The median (solid line), mean (dotted line) and the interquartile range (blue area) for the histogram of the entire period are also shown.
