# Supplementary material for: Have wind turbines in Germany generated electricity as would be expected from the prevailing wind conditions in 2000-2014?
Source: PLoS One. 2019 Feb 6;14(2):e0211028. doi: 10.1371/journal.pone.0211028 (PMC6364903; doi:10.1371/journal.pone.0211028)
Supplement: S2 Fig — The frequency refers to the number of grid cells of the data set showing the trend of a given magnitude. The median (solid line), mean (dotted line) and the interquartile range (blue area) for the histogram of the entire period are also shown. At top, the cumulative distribution function is shown. (PDF) [file pone.0211028.s002.pdf]

Supplementary Material to:

Have wind turbines in Germany generated electricity as would be expected from the prevailing wind conditions in 2000-2014?

Sonja Germer, Axel Kleidon

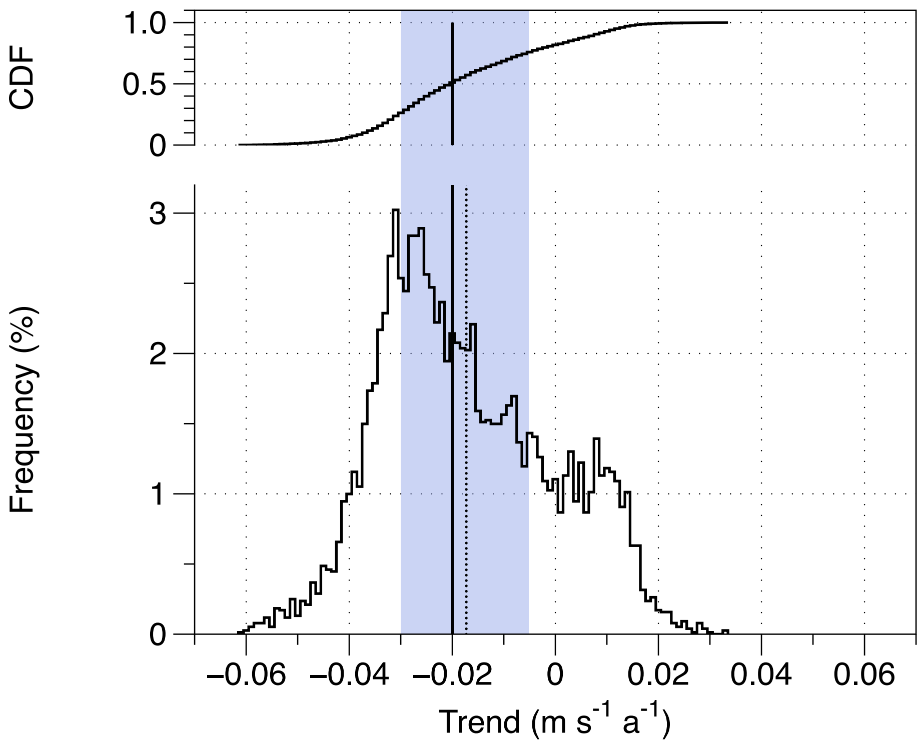

S2 Fig. Histogram of the trend in mean wind speeds using hourly wind speeds in Germany for the years 2000 to 2014 extracted from the COSMO-REA6 dataset at 100 m height. The frequency refers to the number of grid cells of the data set showing the trend of a given magnitude. The median (solid line), mean (dotted line) and the interquartile range (blue area) for the histogram of the entire period are also shown. At top, the cumulative distribution function is shown.
