# Supplementary material for: Have wind turbines in Germany generated electricity as would be expected from the prevailing wind conditions in 2000-2014?
Source: PLoS One. 2019 Feb 6;14(2):e0211028. doi: 10.1371/journal.pone.0211028 (PMC6364903; doi:10.1371/journal.pone.0211028)
Supplement: S3 Fig — (2013). The median (solid line), mean (dotted line) and the interquartile range (blue area) are also shown. (PDF) [file pone.0211028.s003.pdf]

Supplementary Material to:

Have wind turbines in Germany generated electricity as would be expected from the prevailing wind conditions in 2000-2014?

Sonja Germer, Axel Kleidon

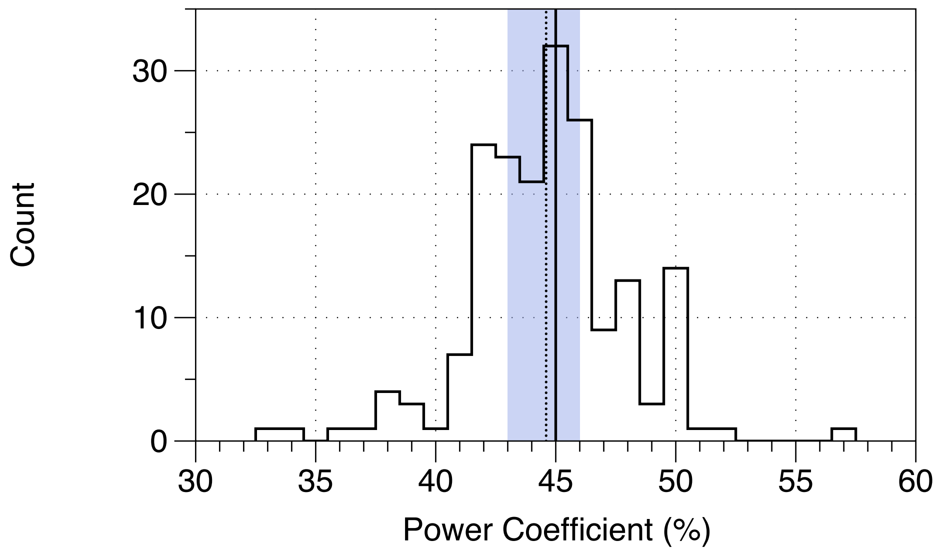

S3 Fig. Distribution of power coefficients taken from the turbine data provided in the review of Carrillo et al. (2013). The median (solid line), mean (dotted line) and the interquartile range (blue area) are also shown.
