# Supplementary material for: Have wind turbines in Germany generated electricity as would be expected from the prevailing wind conditions in 2000-2014?
Source: PLoS One. 2019 Feb 6;14(2):e0211028. doi: 10.1371/journal.pone.0211028 (PMC6364903; doi:10.1371/journal.pone.0211028)
Supplement: S1 Table — (PDF) [file pone.0211028.s005.pdf]

Supporting Information to:

Have wind turbines in Germany generated electricity as would be expected from the prevailing wind conditions in 2000-2014?

Sonja Germer, Axel Kleidon

S1 Table. Values of age distribution shown in Figure 3a.

Year	Mean	5th percentile	25th percentile	Median	75th percentile	95th percentile
2000	3,78	0,33	1,33	3,50	5,67	8,75
2001	4,04	0,33	1,58	3,50	6,25	9,50
2002	4,20	0,33	1,50	3,42	6,67	10,17
2003	4,51	0,42	1,67	3,75	7,08	10,92
2004	4,98	0,58	2,17	4,17	7,50	11,58
2005	5,59	0,83	2,75	4,83	8,17	12,42
2006	6,14	0,75	3,33	5,42	8,67	13,17
2007	6,73	0,92	3,83	6,08	9,33	14,00
2008	7,40	1,17	4,42	6,83	10,08	14,92
2009	8,05	1,17	5,00	7,67	10,83	15,83
2010	8,71	1,33	5,42	8,42	11,58	16,75
2011	9,34	1,50	5,83	9,25	12,33	17,58
2012	9,89	1,25	6,25	10,00	13,17	18,50
2013	10,42	1,25	6,58	10,75	13,92	19,33
2014	10,75	0,83	6,42	11,42	14,67	20,17
