# Supplementary material for: Have wind turbines in Germany generated electricity as would be expected from the prevailing wind conditions in 2000-2014?
Source: PLoS One. 2019 Feb 6;14(2):e0211028. doi: 10.1371/journal.pone.0211028 (PMC6364903; doi:10.1371/journal.pone.0211028)
Supplement: S2 Table — (PDF) [file pone.0211028.s006.pdf]

Supporting Information to:

Have wind turbines in Germany generated electricity as would be expected from the prevailing wind conditions in 2000-2014?

Sonja Germer, Axel Kleidon

S2 Table. Values of installed capacity distribution shown in Figure 3b.

Year	Mean	5th percentile	25th percentile	Median	75th percentile	95th percentile
2000	610,89	80	450	500	600	1500
2001	706,61	80	500	600	1000	1650
2002	820,96	100	500	600	1300	1800
2003	914,62	150	500	600	1500	1800
2004	985,69	150	500	750	1500	2000
2005	1036,34	150	600	1000	1500	2000
2006	1089,53	150	600	1000	1500	2000
2007	1138,05	150	600	1000	1650	2000
2008	1173,98	200	600	1300	1800	2000
2009	1207,41	200	600	1300	1800	2000
2010	1238,98	200	600	1500	1800	2000
2011	1274,28	225	600	1500	2000	2300
2012	1321,90	225	600	1500	2000	2300
2013	1375,08	250	600	1500	2000	2300
2014	1452,82	250	600	1500	2000	2500
