# Supplementary material for: Have wind turbines in Germany generated electricity as would be expected from the prevailing wind conditions in 2000-2014?
Source: PLoS One. 2019 Feb 6;14(2):e0211028. doi: 10.1371/journal.pone.0211028 (PMC6364903; doi:10.1371/journal.pone.0211028)
Supplement: S3 Table — (PDF) [file pone.0211028.s007.pdf]

Supporting Information to:

Have wind turbines in Germany generated electricity as would be expected from the prevailing wind conditions in 2000-2014?

Sonja Germer, Axel Kleidon

S3 Table. Values of rotor swept area distribution shown in Figure 3c.

Year	Mean	5th percentile	25th percentile	Median	75th percentile	95th percentile
2000	1512,54	254	1075	1320	1735	3421
2001	1740,38	314	1276	1466	2290	3848
2002	2025,51	346	1276	1521	3019	4072
2003	2269,63	415	1276	1735	3421	4657
2004	2454,86	452	1276	2124	3848	5027
2005	2585,38	491	1452	2290	3848	5027
2006	2727,87	491	1452	2697	3848	5027
2007	2855,27	531	1466	2827	3959	5281
2008	2952,08	531	1521	3019	3959	5333
2009	3043,80	573	1521	3019	3959	6362
2010	3124,73	573	1521	3217	4657	6362
2011	3215,00	573	1521	3421	4657	6362
2012	3339,98	573	1521	3421	4657	6362
2013	3491,25	661	1662	3848	5027	6362
2014	3741,69	693	1735	3848	5027	8012
