# Supplementary material for: Have wind turbines in Germany generated electricity as would be expected from the prevailing wind conditions in 2000-2014?
Source: PLoS One. 2019 Feb 6;14(2):e0211028. doi: 10.1371/journal.pone.0211028 (PMC6364903; doi:10.1371/journal.pone.0211028)
Supplement: S5 Table — (PDF) [file pone.0211028.s009.pdf]

Supporting Information to:

Have wind turbines in Germany generated electricity as would be expected from the prevailing wind conditions in 2000-2014?

Sonja Germer, Axel Kleidon

S5 Table. Values of park size (N) distribution shown in Figure 3e.

Year	Mean	5th percentile	25th percentile	Median	75th percentile	95th percentile
2000	2,44	1	1	1	2	8
2001	2,60	1	1	1	3	9
2002	2,72	1	1	1	3	9
2003	2,78	1	1	1	3	10
2004	2,83	1	1	1	3	10
2005	2,89	1	1	1	3	10
2006	2,94	1	1	1	3	10
2007	2,97	1	1	1	3	10
2008	3,01	1	1	2	3	10
2009	3,04	1	1	2	3	10
2010	3,04	1	1	2	3	10
2011	3,05	1	1	2	3	10
2012	3,08	1	1	2	3	10
2013	3,10	1	1	2	4	10
2014	3,13	1	1	2	4	10
