# Supplementary material for: Have wind turbines in Germany generated electricity as would be expected from the prevailing wind conditions in 2000-2014?
Source: PLoS One. 2019 Feb 6;14(2):e0211028. doi: 10.1371/journal.pone.0211028 (PMC6364903; doi:10.1371/journal.pone.0211028)
Supplement: S6 Table — (PDF) [file pone.0211028.s010.pdf]

Supporting Information to:

Have wind turbines in Germany generated electricity as would be expected from the prevailing wind conditions in 2000-2014?

Sonja Germer, Axel Kleidon

S6 Table. Values of installed capacity (kW/km²) distribution shown in Figure 3f.

Year	Mean	5th percentile	25th percentile	Median	75th percentile	95th percentile
2000	72,79	1,12	6,02	21,15	67,25	294,60
2001	91,10	1,36	8,61	30,83	87,60	371,58
2002	115,73	1,83	13,79	45,98	117,94	449,26
2003	135,61	2,17	16,92	55,91	141,28	513,62
2004	146,53	2,41	19,67	64,34	156,82	542,14
2005	158,63	2,67	21,55	69,19	175,05	563,42
2006	172,19	2,71	24,65	75,35	192,76	611,36
2007	182,71	2,78	27,16	80,89	201,88	653,20
2008	192,79	2,94	29,20	85,99	212,94	698,15
2009	202,26	2,97	30,67	91,88	222,78	716,69
2010	212,96	3,01	33,19	95,12	231,07	766,32
2011	221,97	3,08	35,54	99,82	246,63	806,77
2012	235,55	3,18	37,40	107,69	260,82	844,98
2013	252,23	3,44	39,64	116,82	277,33	878,70
2014	280,67	3,72	45,74	133,41	309,47	1006,74
